# Osteosarcoma associated with cemento-osseous dysplasia: co-incidence or two related entities?

**DOI:** 10.1007/s11282-024-00758-2

**Published:** 2024-06-09

**Authors:** Suvarna Indermun, Fadi Titinchi, Julandi Alwan, Jean Morkel, Christoffel Johannes Nortje

**Affiliations:** 1 Department of Craniofacial Biology, Pathology and Radiology, Faculty of Dentistry and WHO Collaborating Centre, University of the Western Cape, Cape Town, South Africa; 2 Department of Maxillo-Facial and Oral Surgery, Faculty of Dentistry and WHO Collaborating Centre, University of the Western Cape, Cape Town, South Africa; 3 Department of Anatomical Pathology, National Health Laboratory Service, Tygerberg Hospital, Cape Town, South Africa

**Keywords:** Osteosarcoma, Cemento-osseous dysplasia, Mandible, Cone beam computed tomography (CBCT), Computed tomography (CT)

## Abstract

**Background:**

Osteosarcoma of the jaws is a rare primary malignant tumor of bone. The clinical, radiological and histopathological features of a case associated with cemento-osseous dysplasia is presented.

**Case Report:**

A 57-year-old mixed-race female presented with a large, progressive, swelling of the right mandible. Radiographic examination revealed two associated lesions. Partially defined irregular radiopacities were noted in the left mandible, extending from the premolar to the molar region. The lesion had a cotton-wool appearance and resembled a fibro-osseous lesion; i.e. cemento-osseous dysplasia. A second large, expansive and irregular, radiopaque lesion was noted on the right angle of the mandible, extending beyond the inferior cortex of the mandible. The internal structure was heterogeneous and resembled irregular bone formation. The classic “sunburst” appearance of radiating bony spicules can be seen in the posterior–anterior view and the CBCT 3D reconstruction, indicating the outgrowth of the tumor matrix. Histopathological exam confirmed a final diagnosis of osteosarcoma closely associated with cemento-osseous dysplasia. The patient underwent a fludeoxyglucose-18 (FDG) positron emission tomography (PET) scan which indicated metastasis in the left lung and increased uptake in the right mandible. Chemotherapy was initially administered with a plan to resect the tumor, however, the patient demised as a result of medical complications.

**Conclusion:**

The question in the literature remains whether these two entities are coincidentally found or arise from each other. Nevertheless, it is important for clinicians to closely monitor patients with cemento-osseous dysplasia and biopsy any suspicious lesions that may develop into osteosarcoma.

## Introduction

Fibro-osseous lesions (FOLs) refer to a group of conditions with shared histological features of irregular immature bone surrounded by a collagenous matrix. According to the latest 2022 World Health Organisation (WHO) classification, FOLs include the following: fibrous dysplasia, cemento-osseous dysplasia (COD), familial gigantiform cementoma, segmental odontomaxillary dysplasia, psammomatoid ossifying fibroma and juvenile trabecular ossifying fibroma [1].

COD is the most common benign fibro-osseous lesion (FOL) of the jaws and is generally considered to be self-limiting [1, 2]. As the name suggests, COD is of odontogenic origin and is limited to the tooth-bearing areas of the jaws. CODs embody a spectrum of related lesions that are said to arise from the periodontal ligament [3]. While all variants of COD represent the same histopathological process, they are distinguished based on their clinical and radiographic features. There are three subtypes based on anatomical location and jawbone involvement: periapical, focal and florid [2, 3].

COD has a predilection for middle-aged women of African descent [2, 4, 5]. Radiologically, lesions undergo three stages: lucent, mixed and opaque depending on the progression of mineralization. A “cotton wool” appearance is frequently reported [2, 4, 5]. Typically, COD is asymptomatic, can be diagnosed radiologically and does not require histological confirmation or specific treatment. Some have reported biopsy to be contraindicated as it results in complications such as persistent local infection. In most instances, surgical removal is warranted in secondarily infected lesions and those exposed to the oral environment [2, 3, 6].

In contrast, osteosarcoma (OS) of the jaws is a rare malignant tumor of bone, accounting for only 6% of all OS [1, 7]. OS is characterised by neoplastic cells producing osteoid or bone. One of the most characteristic symptoms of OS is paraesthesia, pain and swelling of the jaw [8, 9]. Radiologically, OS is described as an ill-defined, osteolytic, and ragged radiolucency. It can also result in new periosteal bone formation, perpendicular to the existing cortical plate known as the “sunburst/sunray appearance” [4, 6, 9]. It is interesting to note that OS can arise within FOLs such as Paget’s disease and fibrous dysplasia and in persons exposed to radio therapy [10].

The association of OS with COD is rare. This case report represents a possible association between benign and malignant entities. The literature regarding clinicopathologic features, differential diagnosis and management is also reviewed. Before detailing the clinical presentation and treatment, we confirm that signed informed consent was obtained from the individual participant for whom identifying information is included in this article.

## Case report

**Fig. 1:**
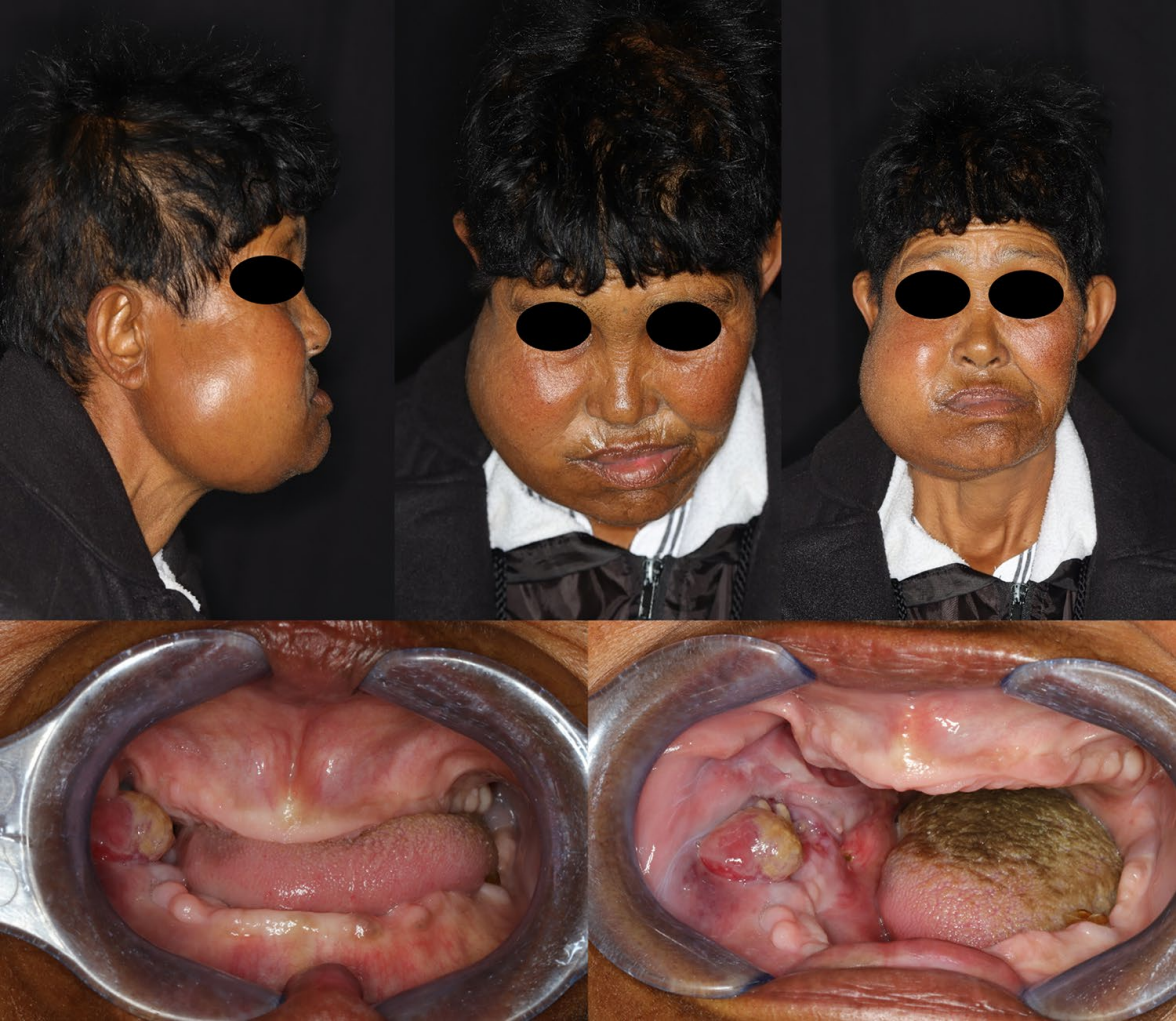
Top; Extra-oral images show large, expansile swelling at the right angle of the mandible. Bottom; Intra-oral images show yellow, ulcerated swelling overlying the right mandibular ridge

A 57-year-old mixed-race female was referred to the Oral and Maxillofacial Surgery outpatient clinic. She presented with the main complaint of a large outgrowth in her mouth with a history of approximately 2 months (Fig. 1). The patient had root remnants removed in this region 2 months before the swelling started. According to the patient, it was increasing in size, painful and interfering with daily oral activities. Apart from a positive history of hypertension and rheumatoid arthritis, the medical history revealed no other medical conditions or allergies. Upon clinical examination, a large palpable facial swelling on the right side of the mandible was noted and no lymphadenopathy was detected.

### Radiology

**Fig. 2:**
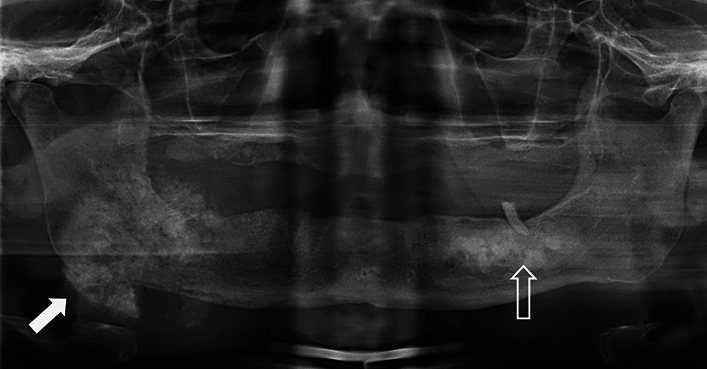
DPR shows partially defined irregular radiopacities in the left mandible within the premolar-molar region. The lesion displays a cotton-wool appearance (open arrow). A large expansive and irregular radiopaque lesion was noted on the right angle of the mandible, extending beyond the inferior cortex of the mandible (solid white arrow)

**Fig. 3:**
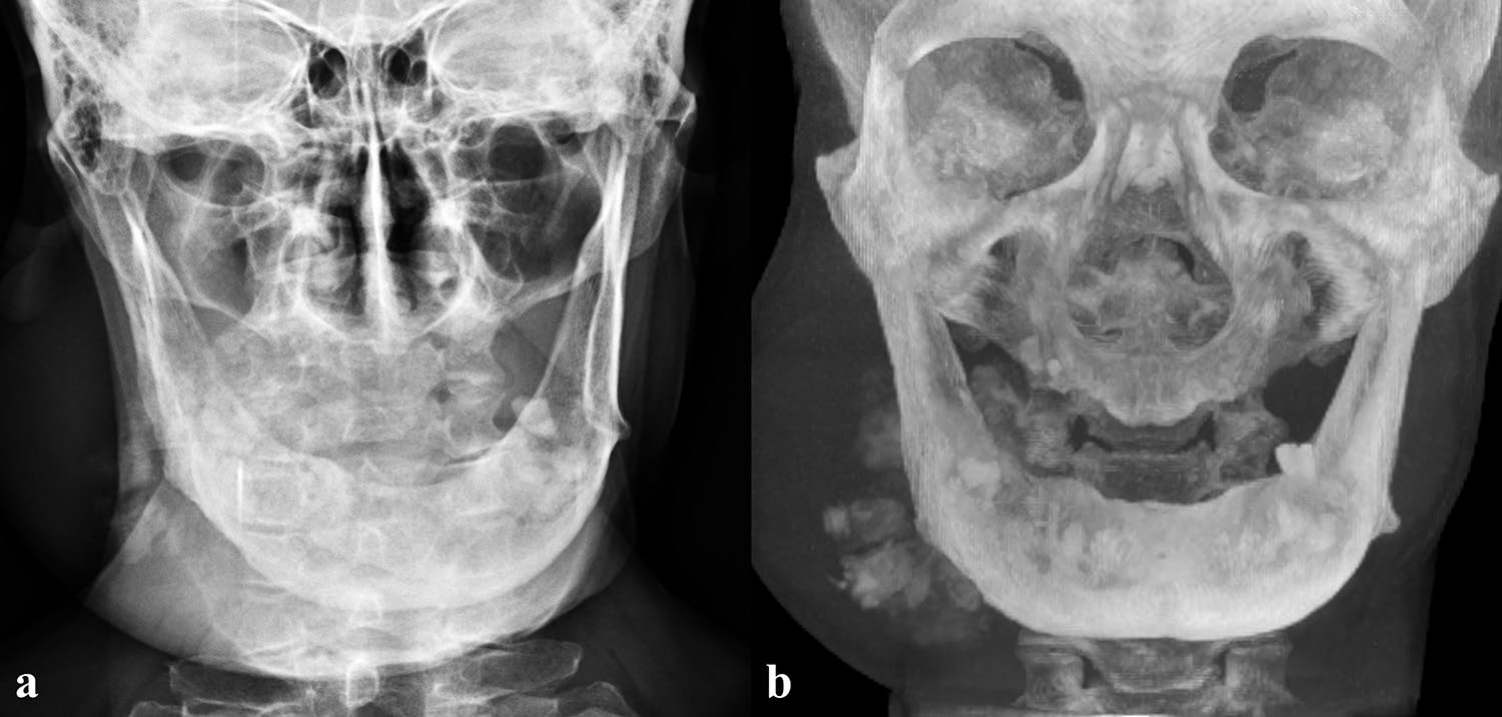
**a** Posterior–anterior mandible shows a sunray appearance at the right body of the mandible and a soft tissue outline of the swelling. **b** CBCT maximum intensity projection shows osseous internal structure of the lesion

A digital panoramic radiograph (DPR) and a posterior-anterior (PA) skull radiograph (Figs. 2, 3) showed two coinciding lesions in the mandible. Partially defined irregular radiopacities were noted in the 3rd quadrant; extending from the premolar to the molar region. The lesion had a cotton-wool appearance. Radiologically, the lesion resembled a fibro-osseous nature; i.e. COD.

**Fig. 4:**
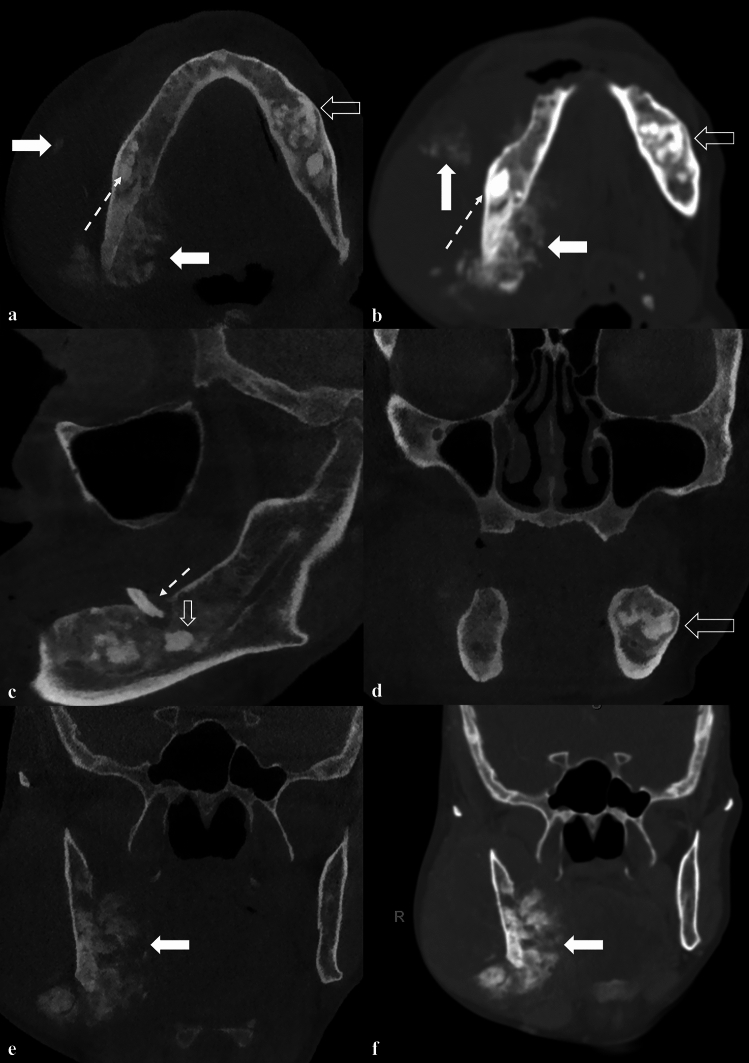
**a** CBCT axial slice, **b** CT axial slice; shows bone formation within the lesion at the right mandible (solid arrow). A cotton wool appearance is seen in the right mandible (dashed arrow), closely related to the OS. A mixed density is seen in the left mandible (open arrow). **c** CBCT sagittal oblique slice shows a root remnant in the left mandible with a periapical low-density (dashed arrow); inferiorly there is a mixed heterogenous lesion. **d** CBCT coronal slice shows an intermediate heterogenous lesion in the left mandible causing lateral expansion (solid arrow). **e** CBCT coronal slice, **f** CT coronal slice; bone formation radiation from the lingual aspect of the right mandible; buccal soft tissue swelling also evident (solid arrows)

**Fig. 5:**
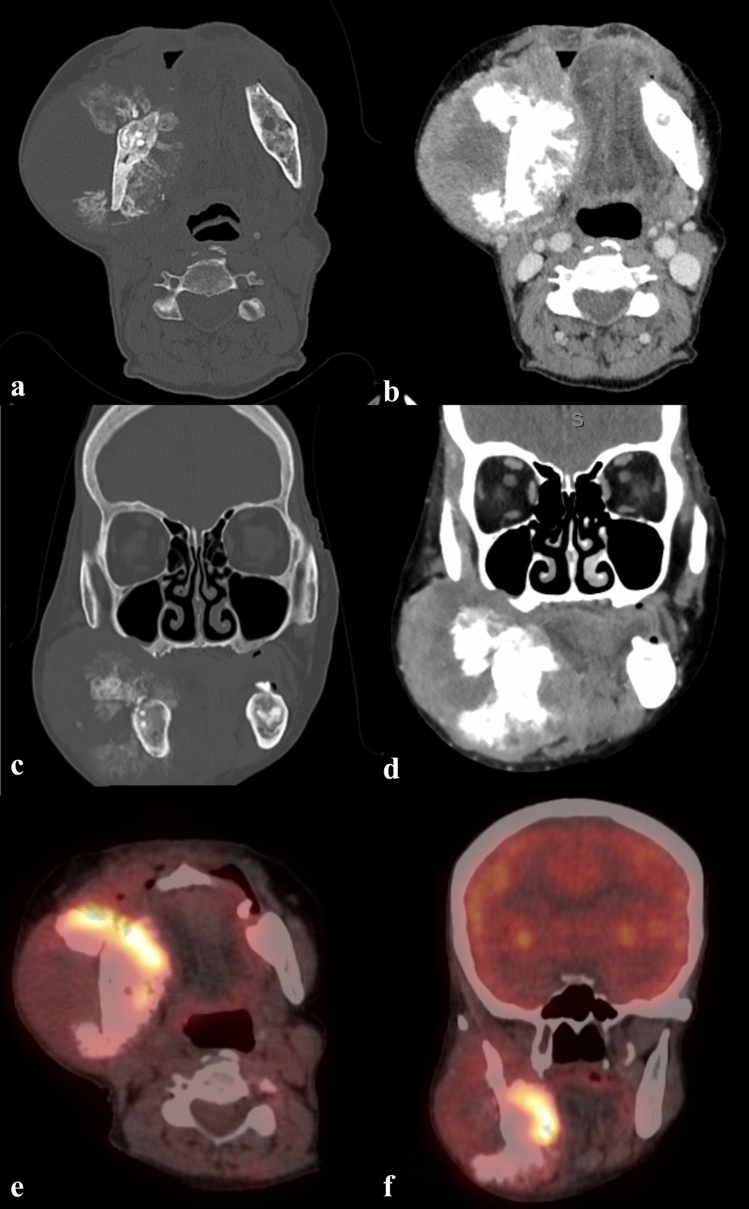
**a** CT axial slice, **b** CT axial slice with contrast, **c** CT coronal slice, **d** CT coronal slice with contras. **a–d**: The sclerotic bone lesion has a sunburst appearance arising from the inner cortex of the mandible with associated heterogeneously enhancing mass which extends to the masticator, buccal and carotid spaces. There is erosion of the posterior wall of the right maxillary sinus. Mass effect on the tongue displacing the tongue to the left. **e** PET scan—axial, **f** PET scan—coronal. **e–f**: Increased uptake noted in the anterior and medial aspect of the lesion

A second large, expansive and irregular, radiopaque lesion was noted on the right angle of the mandible, extending beyond the inferior cortex of the mandible. The borders were poorly defined. The internal structure was heterogeneous/mixed and appeared to resemble bone formation. The inferior alveolar canal was partially traceable on this view. A CBCT and computed tomography (CT) scans were performed, and the volumes were evaluated in all three planes (Fig. 4). Contrast CT and positron emission tomography (PET) scans were also performed showing the extent of the lesion and increased uptake in the right mandible, respectively (Fig. 5). A sunburst appearance of radiating bony spicules and accompanying soft tissue swelling can be seen indicating outgrowth of the tumor matrix.

Specifically for this case, the differential diagnosis included the association of a malignant and benign lesion; namely OS, with a FOL, i.e. COD. The diagnosis of COD was made radiologically only.

### Histopathology

**Fig. 6:**
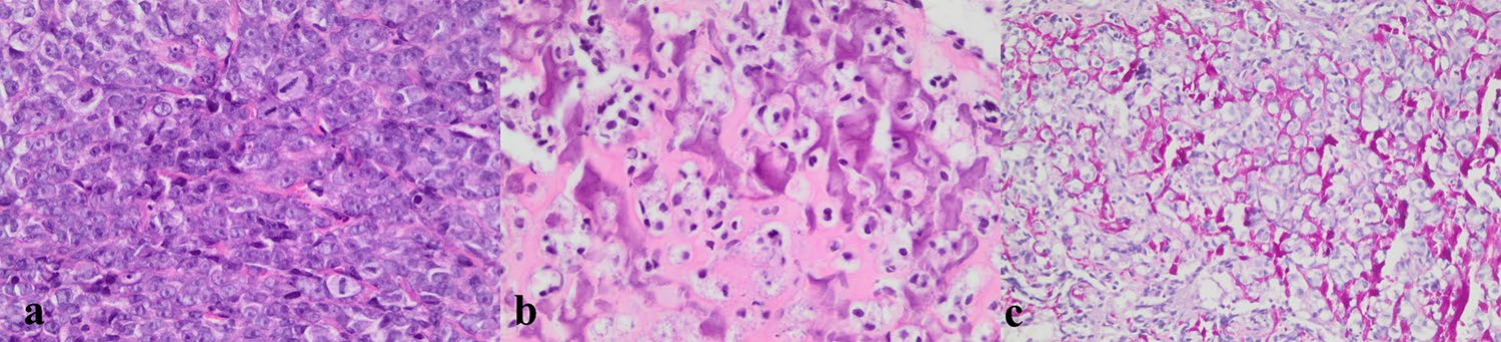
**a** High magnification demonstrating malignant epithelioid cells arranged in lobules, with hyperchromatic nuclei and prominent nucleoli. Prominent mitotic figures are also demonstrated (× 400) **b** Lace-like malignant osteoid at high magnification (× 400), **c** Periodic-Acid Schiff highlighting the lace-like osteoid (× 200)

An incisional biopsy revealed a malignant neoplasm with epithelioid to oval cells arranged in sheets and lobules. Nuclear features varied from hyperchromatic to vesicular with prominent nucleoli. The cytoplasm was eosinophilic to clear, with irregular cell borders, in a background of variable amounts of immature osteoid deposition and frequent mitoses (Fig. 6). Necrosis in this biopsy specimen was absent. Immunohistochemistry (SATB2) supported the diagnosis of an OS, after other malignant tumors, including carcinomas and lymphomas, were excluded.

### Management

The patient underwent fludeoxyglucose-18 (FDG) PET scan which showed perineural invasion of the right inferior alveolar nerve as well as pulmonary nodules in the left upper lobe and left supraclavicular lymph node. Upon review of the patient at the multidisciplinary meeting, the treatment plan involved the administration of neoadjuvant chemotherapy with surgical resection of the tumor. The patient received three doses of chemotherapy and then demised from medical complications prior to receiving any surgical intervention.

## Discussion

FOLs of the craniomaxillofacial region are described as the replacement of bone by cellular fibrous tissue containing varied amount of mineralized tissue. The lesions share a similar histological appearance, thus clinical and radiological features must be correlated to determine a final diagnosis [7, 11]. FOLs usually present a favourable prognosis, but evidence suggests that some FOLs, especially fibrous dysplasia, may have a risk of spontaneous malignant transformation [11].

COD is the most common FOL and has a predilection for middle-aged women of African descent [3, 4, 9]. As the name suggests, it is limited to the tooth-bearing region; characterized by the replacement of bone with cementum-like material [3, 6, 7]. These lesions lack expansile growth, are asymptomatic and normally detected as incidental findings on routine radiographic examination [2].

COD is further divided into three categories: periapical cemental dysplasia (PCD), focal COD and florid COD. PCD is a localized variant affecting the apices of vital mandibular anterior teeth. The focal variant is a solitary lesion usually not associated with a tooth and florid COD involves two or more quadrants of the jaws [5, 9].

Radiologically, as COD matures it transforms between three stages depending on the degree of mineralization, namely: (1) lucent/early stage where normal bone is resorbed, (2) mixed stage followed by (3) the opaque/final phase. New bone is deposited in increasing amounts in the second and third stages [5, 9, 12].

The interpretation of COD can be made based on the radiologic features and clinical findings [13]. Since COD can be diagnosed radiologically, it does not always require histological confirmation [2]. Due to the decreased vascularity in COD, biopsies are controversial as they predisposes the patient to osteomyelitis [2, 3, 14, 15]. CODs usually do not require treatment, but due to the tendency for mature lesions to become secondarily infected or exposed in the oral cavity, surgical intervention is then required to avoid complications such as osteomyelitis or draining cutaneous fistula [13].

Turning to OS, which in contrast to COD, is a malignant tumor of bone with a sinister prognosis. Symptoms such as pain and swelling are characteristic [6]. The case reported by Schneider et al. was the first example of a sarcoma arising in association with florid osseous dysplasia [15]. The concurrence of COD and OS is rare and, therefore, sparsely reported in the literature. There are only six reported cases known to the authors. A review of these cases was conducted, and a comparison is summarized in Table 1 [2, 6, 15–17]. The concurrence of COD and OS, as noted in these cases, prompts a re-evaluation of potential pathophysiological links. Wagner et al. has suggested that certain fibro-osseous lesions may harbour malignant potential under specific conditions [11]. Furthermore, genetic studies have identified pathways that could be implicated in both COD and OS, suggesting a relationship that may extend beyond mere coincidence [10]. The cases outlined in Table 1 demonstrate a range of demographic profiles and clinical presentations, yet consistently show an association between COD and the subsequent development of OS in the mandible. For instance, Schneider et al. and Melrose and Handlers both reported cases from the USA where patients presented with clinical and radiographic features suggestive of both COD and OS in the mandible [15, 16]. Lopes et al., Olusanya et al. and Haefliger et al*.*, noted similar findings in Brazil, Nigeria and Switzerland respectively, indicating that this association is observed across different populations and geographic locations [2, 6, 17]. Haefliger et al. presented a case of a patient with established COD who developed high-grade OS over a period of 9 years [2]. This longitudinal evidence supports the hypothesis that COD may, under certain conditions, undergo malignant transformation. The review of these literatures supports a hypothesis of related pathogenesis, warranting further molecular and epidemiological studies.

**Table 1:** Studies reporting cases of COD with malignant transformation

Authors, Year	Case	Country	Age	Gender	Race	Site	Radiographic features	Clinical features	Histology
Schneider et al*.*, 1999	1	USA	54	Female	Black	Malignancy: Left mandible COD: Right mandible	Left mandible: Predominantly radiolucent are in the left mandible, multiple small opaque foci present internally, loss of superior cortex Right mandible: well-defined opaque lesion in area of missing molar	Numbness of lower left lip after extraction of loose molar, buccal expansion of the left body of the mandible	Confirmed malignant fibrous histiocytoma with benign fibro-osseous lesion
Melrose and Handlers, 2003	2	USA	36	Female	Black	Malignancy: Left mandible COD: bilateral mandible	Diffuse ill-defined radiopacity with buccal and lingual expansion Previous radiographs (3 years earlier) showed bilateral radiopacities of the alveolar process of the maxilla and mandible	Marked swelling of the left face, no paraesthesia	Confirmed high-grade OS Microscopic changes compatible with COD noted
Lopes et al., 2010	3	Brazil	44	Female	Black	Right mandible	ill-defined margin, increased bony sclerosis, patchy bone destruction, new bone formation, and periosteal reaction with a Codman’s triangle Additional many other mixed lesions in jaws	swelling of the posterior right mandibular alveolar ridge, extending to the buccal sulcus was observed. Two molars had recently been extracted and the premolar was displaced	Confirmed high-grade OS Microscopic changes compatible with COD noted
Olusanya et al*.,* 2012	4	Nigeria	65	Female	Not specified	Left mandible	Left mandible: Osteolytic lesion with radiopaque spikes Right mandible: irregular apical opacities	Swelling, spontaneous origin, painful intermittently, mobile teeth, buccal and lingual sulcus obliterate by tumor mass	Confirmed OS and COD
	5	Nigeria	45	Female	Not specified	Right mandible	Right mandible: bone destruction Left mandible: periapical radiopacities extending the body of the mandible	swelling of spontaneous origin, no remarkable symptoms, but was increasing progressively in size	Confirmed chondroblastic OS
Haefliger et al*.*, 2021	6	Switzerland	30	Female	Caucasian	COD: Bilateral mandible Malignancy: left mandible	COD: Expansile, opaque lesions in the posterior regions of all 4 quadrants; no periosteal reaction or signs of locally aggressive behaviour, root resorption noted in the mandible 9 years later: cortical erosion, enlarged lytic areas with progressive root resorption	Bilateral mucosal swellings of the mandible; 9 years later patient reported increasing swelling and pain in the left mandible;	Confirmed COD; high-grade OS diagnosed after 9 years; confirmed Li-Fraumeni syndrome

*COD c*emento-osseous dysplasia, *OS* osteosarcoma

OS of the jaws is rare and clinically distinct from OS of the long bones [2, 18], with the latter being diagnosed amongst adolescents and the former presenting between the 4th–5th decades [9]. Males and females are said to be equally affected. Paraesthesia, pain, loosening of teeth, swelling and ulceration are the most common symptoms present. Previous radiation therapy, fibrous dysplasia, Paget's disease are known pre-disposing factors [9, 12].

OS presents radiographically as an osteolytic lesion. Mineralization of the tumor matrix is frequently seen and depending on the stage of mineralization, it can appear radiolucent, mixed, or radiopaque. Several periosteal reactions have been described in the literature. These include Codman’s triangle; seen at the periphery of elevated periosteum, and the characteristic “sunburst” appearance [4, 9, 12, 19]. These features are seen in mandibular tumors [9]**.**

### Pathogenesis and histopathology

Many OS arise spontaneously, however, there may be an increased risk of developing secondary OS with prior radiotherapy, an underlying Paget’s disease of bone, pre-existing bone lesions, or genetic predispositions (e.g. Li-Fraumeni syndrome). Molecular alterations include TP53 gene and retinoblastoma gene pathway alterations, translocations and numerical alterations. Cell cycle regulators, including CDK4 and CDKN2A genes, may also be altered in some cases [20]. The jaw bones are more commonly affected in the head and neck than any other craniofacial bones The majority of OS in the gnathic bones occurs intramedullary, but cases of parosteal types have been reported [21].

Conventional OS is characterised by atypical cells producing neoplastic, lace-like osteoid. The cells may be polygonal or epithelioid in the osteoblastic variant, similar to the case described in this report. The matrix may demonstrate extensive variability, including heavy mineralised sclerotic bone. Higher-grade OS are more aggressive, resulting in extensive bone destruction. There are several histologic subtypes, classified according to the predominant matrix, including osteoblastic, chondroblastic and fibroblastic. Immunohistochemical staining to confirm osteoblastic differentiation includes SATB2, MDM2 and CDK4 [21].

To date, and to the authors’ best knowledge, six cases have been reported where patients with COD also developed OS [2, 6]. One of these patients was known with Li-Fraumeni Syndrome, which predisposes those affected to developing malignant tumors due to *TP53* mutations [2]. The molecular pathogenesis of COD is not well known but is not considered a precursor lesion for OS [2].

### Diagnosis and management

Management of any FOLs suspected to have developed malignancy is an urgent incisional biopsy to confirm the diagnosis. Histopathological confirmed malignancies should be discussed in a multi-disciplinary tumor board to establish the best care for the patient. OS are predominantly managed with an initial period of chemotherapy followed by wide surgical resection unless the patient is deemed more suitable for palliation [18].

Wagner et al*.*, performed a systematic review of the literature concerning all documented cases of malignant transformation of craniomaxillofacial fibro‐osseous lesions [11]. Their results revealed that fibrous dysplasia was the only FOL that seemed to have a significant risk of malignant transformation; with OS being the most common malignant tumor [11]. It is advised that the initial diagnosis of any FOL be substantiated by clinical, radiological, and histopathological evaluation since some cases can progress to OS or lead to a misdiagnosis [11].

The association of OS and COD raises the question of whether a collision tumor has resulted in the transformation of COD into OS or their simultaneous presence is a mere coincidence [6, 15]. There is no consensus in the literature about the potential for malignant transformation of COD. Based on the literature reviewed, and this individual case, we cannot conclusively establish causation, but the co-occurrence of these conditions suggests more than a coincidental relationship. It highlights a potential pathogenic link, warranting further investigation. Whilst this paucity exists in the literature and the risk of malignant transformation may be even less, it should not be considered negligible. Awareness of possible malignant transformation can guide healthcare professionals to establish more stringent follow-ups. This is especially true for lesions such as COD as it is rarely surgically treated.
